# Population-specific patterns of IgE sensitization in Northwest China

**DOI:** 10.1016/j.clinsp.2026.101121

**Published:** 2026-09-08

**Authors:** Jiahao Guan, Fei Wang, Zitong Wang, Yihan Dong, Jun Wang, Yajuan Ren, Zifan Lu, Lixia Zhang

**Affiliations:** a Department of Clinical Laboratory, Shaanxi Provincial People′s Hospital, Xi′an, Shaanxi, PR China; b Medical College, Yan'an University, Yan'an, Shaanxi, PR China; c Department of Respiratory Medicine Ⅰ, Shaanxi Provincial People's Hospital, Xi′an, Shaanxi, PR China; d Department of Respiratory Medicine Ⅱ, Shaanxi Provincial People's Hospital, Xi′an, Shaanxi, PR China; e Translational Medicine Center of Shaanxi Provincial People's Hospital, Xi′an, Shaanxi, PR China

**Keywords:** IgE sensitization, Allergic diseases, China, Epidemiology, Sex difference, Age distribution

## Abstract

**Background:**

Immunoglobulin E (IgE)-mediated allergic diseases are a major and growing global health burden, with sensitization patterns varying by region and population. Comprehensive data from Northwest China remain limited. This study aimed to characterize population-specific IgE sensitization profiles in this region and explore their associations with sex and age.

**Methods:**

We retrospectively analyzed 13,123 patients with allergic diseases at a large tertiary center in Northwest China from January 2017 to June 2024. Demographic data, clinical diagnoses, and serum allergen-specific IgE (sIgE) levels measured by ImmunoCAP were collected. Sensitization was defined as sIgE ≥ 0.35 kUA/L. Statistical analyses included Chi-Square tests and Spearman correlation.

**Results:**

The cohort included 5,928 males (45.18%) and 7,195 females (54.82%), with children under 5-years constituting the largest subgroup (23.04%). Males had significantly higher sensitization rates than females for both food allergens (p < 0.001) and inhalant allergens (p < 0.001). Food allergen sensitization peaked in children under 10-years (6.69%–8.22%), while inhalant allergen sensitization peaked in the 10–20 years age group (10.92%). Among tested allergens, peanut induced the highest reactivity level, and egg white showed the highest disease-specific sensitization rate in asthma patients (19.57%).

**Conclusions:**

This study reveals distinct, population-specific IgE sensitization patterns in Northwest China, significantly influenced by sex and age. Findings highlight a higher sensitization burden in males and an age-dependent shift from food to inhalant allergen predominance. These insights support a stratified, precision-based approach to allergy diagnosis and management tailored to regional allergen exposures.

## Highlights

• The allergic rate of males was higher than that of females.
• With increasing age, allergens turned from food to inhalation.
• Nearly two thirds of allergic patients have no specific IgE and need to be tested for allergens regionally.

## Introduction

Allergic diseases, including asthma, urticaria, and allergic rhinitis, represent a significant and growing public health burden worldwide.^1^, ^2^, ^3^ These conditions, driven by IgE-mediated hypersensitivity to common environmental and food allergens, impair quality of life and incur substantial socioeconomic costs.^4^^,^^5^ The prevalence of allergic diseases has markedly increased in recent decades, particularly among children and adolescents. This trend has attracted significant attention and correlates with rapid urbanization and dietary changes, suggesting the involvement of gene-environment interactions.^5^, ^6^, ^7^

The comprehensive characterization of an allergen includes an analysis of its IgE-binding properties and confirmation of its clinical relevance.^8^ A critical aspect of understanding and managing allergic diseases is the characterization of allergen sensitization patterns. Epidemiological studies consistently demonstrate that sensitization profiles are not uniform; rather, they are significantly influenced by factors such as age, sex, geographic location, and seasonal exposure.^5^^,^^9^, ^10^, ^11^ Food sensitization is often more prevalent during early childhood, whereas reactivity to aeroallergens may increase or persist into adulthood.^11^^,^^12^ Furthermore, the prevalence of allergies to various tree nuts appears to vary significantly across different regions worldwide.^13^, ^14^, ^15^, ^16^ Furthermore, sex-based differences in immune function can lead to variations in the prevalence and manifestation of allergies throughout the lifespan.^17^ Internationally, large collaborative studies, such as the GA2LEN (Global Asthma and Allergy European Network) survey, have mapped sensitization patterns across Europe, revealing significant geographic gradients.^18^ However, comparable comprehensive data from China, characterized by its unique climate, vegetation, and urbanization patterns, remain scarce. Most studies conducted in China to date are constrained by smaller sample sizes or shorter timeframes, which limits their statistical power to adequately examine subgroup differences across multiple seasons adequately.

Given the unique climatic and vegetative characteristics of Northwest China, this study aims to fill a significant gap in the existing literature. To this end, we conducted a large-scale retrospective analysis of clinical data from 13,123 patients diagnosed with allergic diseases over a seven-year period (2017‒2024) in Northwest China. The primary objectives of this study were: 1) To describe the demographic and clinical characteristics of a large cohort of Chinese individuals with allergies; 2) To analyze the prevalence and patterns of sensitization to major food and inhalant allergens; and 3) To evaluate the associations between sensitization profiles and key variables, including sex, age, and specific allergic diseases. By providing a detailed sensitization map, this study aims to inform more personalized diagnostic and public health strategies for allergy management in China.^19^

## Methods

### Study design and setting

This was a retrospective cohort study conducted at the Shaanxi Provincial People’s Hospital, a major tertiary referral center in Northwest China. We analyzed clinical data from patients diagnosed with allergic diseases between January 2017 and June 2024.

### Ethical approval and consent

The study protocol was reviewed and approved by the Ethics Committee of Shaanxi Provincial People’s Hospital (Approval No. [2024] Ethics Review No. R131). As this study involved a retrospective analysis of anonymized clinical data, the requirement for obtaining individual written informed consent was waived in accordance with national ethical guidelines and the specific approval from the ethics committee.

### Inclusion and exclusion criteria

Consecutive patients who visited the allergy, dermatology, respiratory medicine, or pediatric clinics during the study period were screened for eligibility.

Inclusion Criteria: Patients were included if they: 1) Received a clinical diagnosis of one or more allergic diseases (as defined in Section 4) by a board-certified specialist; 2) Underwent serum allergen-specific Immunoglobulin E (sIgE) testing as part of their standard diagnostic evaluation; and 3) Had complete records for demographic data, clinical diagnosis, and *sIgE* results.

Exclusion Criteria: Patients were excluded for: 1) Missing or incomplete core data; 2) Concurrent diagnoses that could confound IgE interpretation, including primary immunodeficiency disorders, active parasitic infections, or hematological malignancies; or 3) Use of systemic immunosuppressants (e.g., oral corticosteroids, biologic agents) at the time of the sIgE blood draw.

A total of 13,123 patients met all inclusion criteria and formed the final study cohort.

### Diagnostic criteria for allergic diseases

All clinical diagnoses were made by relevant board-certified specialists following established international guidelines:

Asthma: Global Initiative for Asthma (GINA) criteria.

Allergic Rhinitis: Allergic Rhinitis and its Impact on Asthma (ARIA) guidelines.

Urticaria: Diagnosed according to the S3 Guideline Urticaria (Part 1: Classification and diagnosis of urticaria), based on the sudden appearance of wheals, angioedema, or both.

Eczema (Atopic Dermatitis): Diagnosed according to Hanifin and Rajka or UK Working Party diagnostic criteria. This category primarily includes endogenous eczema without confirmed IgE-mediated sensitization.

Allergic Dermatitis (IgE-mediated): Diagnosis required eczematous or urticarial lesions with a clinically suspected external allergic trigger, confirmed by positive serum sIgE to relevant allergens (e.g., foods, aeroallergens). Importantly, patients with suspected allergic contact dermatitis (delayed-type hypersensitivity, e.g., to metals, fragrances, or preservatives) confirmed by positive patch testing were strictly excluded from this subgroup, as these conditions are mediated by T cells rather than IgE. This distinction ensures that the allergic dermatitis subgroup specifically reflects IgE-mediated cutaneous hypersensitivity.

Allergic Purpura (IgA Vasculitis): Diagnosed according to the EULAR/PRINTO/PRES classification criteria. Although IgA vasculitis is primarily a type III hypersensitivity (immune complex-mediated) disorder rather than an IgE-mediated condition, it is commonly categorized under allergic diseases in clinical practice and routinely undergoes allergen-specific IgE testing. Therefore, this subgroup was included in the overall cohort to reflect real-world clinical diagnostic patterns, with the understanding that its underlying pathophysiology differs from classical IgE-mediated allergic diseases. This distinction is addressed in the Discussion section.

### Allergen-specific IgE testing: methodology and definitions

#### Assay platform and procedure

Serum allergen-specific IgE (sIgE) levels were quantified using the ImmunoCAP 250 system (Thermo Fisher Scientific/Phadia, Uppsala, Sweden), a standardized and widely validated Fluoroenzyme Immunoassay (FEIA). Testing was performed with commercial reagents according to the manufacturer's instructions.

#### Quantitative reporting and positivity cut-off

Results are reported in kilo-Units of Allergen-specific IgE per Liter (kUA/L). Sensitization was defined using the ImmunoCAP class system:

Class 0 (Negative): < 0.35 kUA/L
Class 1 (Low): 0.35–0.70 kUA/L
Class 2 (Moderate): 0.71–3.50 kUA/L
Class 3 (High): 3.51–17.50 kUA/L
Class 4 (Very High): 17.51–50.00 kUA/L
Class 5 (Very High): 50.01–100.00 kUA/L
Class 6 (Very High): > 100.00 kUA/L

For the primary analysis in this study, a positive sensitization was defined as an *sIgE* level ≥ 0.35 kUA/L (ImmunoCAP Class ≥ 1). Recognizing that higher sIgE levels have greater predictive value for clinically relevant allergy, we additionally performed a sub-analysis defining “higher levels of sensitization” as sIgE ≥ 3.51 kUA/L (ImmunoCAP Class ≥ 3), a threshold commonly used in clinical practice to indicate clinically meaningful IgE-mediated sensitization. Results of this sub-analysis are presented in Table 1 to complement the primary findings.

**Table 1:** Disease-specific higher levels of sensitization patterns for food and inhalation allergens (sIgE ≥ 3.51 kUA/L).

**Disease Category**	**Total Patients (N)**	**Food Allergen Sensitization (≥ 3.51 kUA/L), n (%)**	**Inhalation Allergen Sensitization (≥ 3.51 kUA/L), n (%)**
Asthma	3,454	98 (2.84%)	45 (1.30%)
Allergic Rhinitis	1,925	46 (2.39%)	35 (1.81%)
Urticaria	2,563	48 (1.87%)	29 (1.13%)
Allergic Purpura	2,146	39 (1.82%)	35 (1.63%)
Dermatitis	1,617	22 (1.36%)	17 (1.05%)
Eczema	748	10 (1.34%)	6 (0.80%)
Total	12,453	263 (2.11%)	167 (1.34%)

Note: This table presents data for patients with higher levels of sensitization (sIgE ≥ 3.51 kUA/L). Percentages are calculated based on the total number of patients within each disease category. The total number of patients (12,453) differs from the overall cohort (13,123) as this sub-analysis includes only those with available sIgE test results for the respective allergen categories.

#### Allergen nomenclature

All allergen names reported have been standardized to the specific designations of the ImmunoCAP system. A comprehensive reference list is provided in Supplementary Table S1.

### Data collection and seasonal assignment

Demographic information, clinical diagnoses, consultation dates, and *sIgE* results were extracted from electronic medical records.

### Selection of allergen-specific IgE panel

The allergen-specific IgE tests were performed based on clinical indication. A core panel of allergens was selected for routine screening based on their epidemiological relevance in Northwest China and alignment with Chinese allergy practice guidelines. This panel included common inhalant allergens (e.g., house dust mites, mugwort, Artemisia argyi, cat/dog dander, molds) and food allergens (e.g., egg white, milk, peanut, shrimp, wheat). Testing was not uniform for all allergens across all patients; clinicians requested tests based on individual patient history and symptom profile. Therefore, the number of allergens tested per patient varied.

### Statistical analysis

Data were analyzed using SPSS version 26.0 (IBM Corp., Armonk, NY, USA). Categorical variables are presented as numbers and percentages. Group comparisons were performed using the Chi-Square test or Fisher’s exact test, as appropriate. The Spearman rank correlation coefficient was used for non-parametric correlation analysis. Continuous variables between two groups were compared using the Mann-Whitney *U*-test. A two-sided p-value of less than 0.05 was considered statistically significant.

## Results

### Annual distribution of study participants

A total of 13,123 participants were enrolled in this study from 2017 to 2024. As detailed in Table 2, the annual distribution exhibited substantial variation. Participant enrollment peaked in 2018 at 16.65% (n = 2,185), followed closely by 2023 at 16.18% (n = 2,123). The lowest enrollment occurred in 2017, accounting for 4.34% (n = 570). Participant recruitment remained relatively consistent from 2018 to 2023, with a notable decline in 2024 to 9.01% (n = 1,183).

**Table 2:** Annual distribution of study participants (2017‒2024).

**Year**	**Frequency**	**Percentage**
2017	570	4.34
2018	2185	16.65
2019	2006	15.29
2020	1378	10.50
2021	1973	15.03
2022	1705	12.99
2023	2123	16.18
2024	1183	9.01
Total	13123	100.00

Note: Percentages are calculated as the proportion of participants enrolled in each year out of the total cohort (n = 13,123). Data for 2024 are incomplete, covering only January to June.

### Demographic characteristics of the study population

The cohort comprised 5,928 males (45.18%) and 7,195 females (54.82%). As summarized in Table 3, the age and sex distribution exhibited distinct patterns: the age group under - years constituted the largest proportion (23.04%), with a higher representation of males (30.21% of all males) compared to females (17.12% of all females). Conversely, the age group of 20‒30 years displayed a predominance of female participants (22.82% of all females vs. 10.80% of all males). The proportion of participants gradually decreased with increasing age.

**Table 3:** Demographic characteristics: age and sex distribution of the study population.

**Age Group**	**Male**		**Female**		**Total**	
	**Number**	**%**	**Number**	**%**	**Number**	**%**
< 5	1,791	30.21	1,232	17.12	3,023	23.04
5‒10	1,050	17.71	724	10.06	1,774	13.52
10‒20	659	11.12	625	8.69	1,284	9.78
20‒30	640	10.80	1,642	22.82	2,282	17.39
30‒40	574	9.68	1,078	14.98	1,652	12.59
40‒50	369	6.22	676	9.40	1,045	7.96
50‒60	355	5.99	635	8.83	990	7.54
60‒70	307	5.18	401	5.57	708	5.40
≥ 70	183	3.09	182	2.53	365	2.78
Total	5,928	100	7,195	100	13,123	100

Note: For Male and Female columns, ‘%’ refers to the proportion within that sex subgroup (denominator: total males or total females). The ‘%’ in the Total column refers to the proportion of the total cohort (n = 13,123).

### Disease distribution among study participants

The disease spectrum among the 13,123 study participants is summarized in Table 4. Asthma was the most prevalent condition, affecting 26.32% (n = 3,454) of participants, followed by urticaria at 19.53% (n = 2,563) and allergic purpura at 16.35% (n = 2,146). Allergic rhinitis accounted for 14.67% (n = 1,925) and dermatitis for 12.32% (n = 1,617). Eczema comprised 5.70% (n = 748), and other allergic conditions accounted for 5.11% (n = 670).

**Table 4:** Disease distribution among study participants (n = 13,123).

**Disease**	**Frequency**	**Percentage**
Asthma	3,454	26.32
Urticaria	2,563	19.53
Allergic purpura	2,146	16.35
Allergic rhinitis	1,925	14.67
Dermatitis	1,617	12.32
Eczema	748	5.70
Other	670	5.11
Total	13,123	100.00

Note: Participants could be diagnosed with more than one allergic condition. Percentages indicate the proportion of the total cohort diagnosed with each specific disease.

### Allergen reactivity and positive species detection

**Fig. 1:**
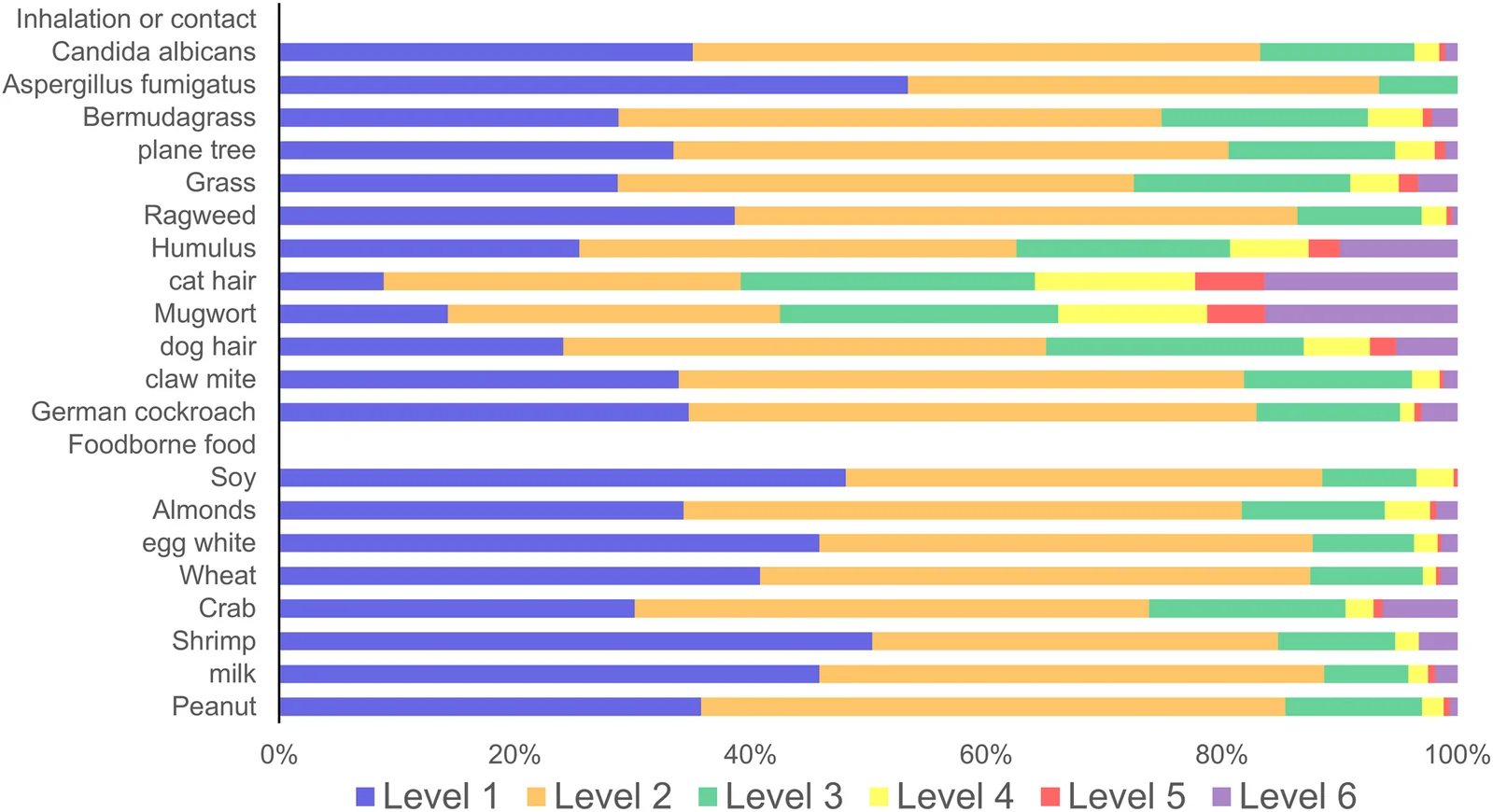
Quantification os allergen-specific IgE reactivity levels in patient sera.

The allergen-specific IgE reactivity profiles were quantitatively assessed across six levels (Levels 1–6), as detailed in Fig. 1. Inhalant allergens, such as *Candida albicans* and *Aspergillus fumigatus*, exhibited moderate reactivity (Level 3–4). Environmental allergens, including *Bermudagrass, Platanus spp., grass, ragweed, Humulus, cat hair, mugwort, dog hair,* and *claw mite*, demonstrated variable reactivity, ranging from Level 2 to Level 4. The allergen from the *German cockroach* showed elevated reactivity (Level 4). Among foodborne allergens, *soy* and *almonds* displayed low reactivity (Level 1–2), while egg white, wheat, crab, shrimp, milk, and peanut elicited higher *IgE* responses, with *peanut* achieving the highest reactivity (Level 5–6). The distribution of positive species detection, as detailed in Table 5, indicated that the majority of participants (63.96%, n = 8,393) exhibited no positive detection. The frequency of positive detections decreased rapidly with an increasing number of detected species.

**Table 5:** Distribution of positive species detection among study participants (n = 13,123).

**Number of Positive Species**	**Frequency**	**Percentage**
0	8,393	63.96%
1	1,999	15.23%
2	953	7.26%
3	507	3.86%
4	362	2.76%
5	244	1.86%
6	156	1.19%
7	94	0.72%
8	91	0.69%
9	78	0.59%
10	82	0.62%
11	58	0.44%
12	52	0.40%
≥13	54	0.41%
Total	13,123	100%

Note: ‘Number of Positive Species’ refers to the count of distinct allergen species with a specific IgE level ≥0.35 kUₐ/L. ‘Percentage’ is calculated based on the total cohort of 13,123 participants.

### Gender-based differences in allergen sensitization

Analysis of allergen sensitization patterns by gender revealed significant differences in prevalence rates. As shown in Table 6, the prevalence of sensitization to food allergens was significantly higher in male patients (47.3%, 2,805/5,928) than in female patients (36.6%, 2,634/7,195) (χ^2^ = 153.592, p < 0.001). Similarly, the prevalence of sensitization to inhalation allergens was also significantly higher in males (75.4%, 4,469/5,928) compared to females (56.1%, 4,033/7,195) (χ^2^ = 532.612, p < 0.001). These findings indicate a consistent pattern of higher allergic sensitization prevalence among males for both major allergen types.

**Table 6:** Gender-based differences in allergen sensitization patterns.

**Allergen Type**	**Male (n = 5,928)**		**Female (n = 7,195)**		**p-value**
	**Positive (n)**	**Prevalence (%)**	**Positive (n)**	**Prevalence (%)**	
Food Allergen	2,805	47.3	2,634	36.6	<0.001
Inhalation Allergen	4,469	75.4	4,033	56.1	<0.001

Note: ‘Prevalence (%)’ refers to the within-group prevalence, calculated as (Number of Positive Cases within the sex subgroup / Total Number in that sex subgroup) × 100%. For example, the food allergen sensitization prevalence among all males is 47.3% (2,805/5,928).

### Temporal trends in sensitization

The temporal trends in allergen sensitization from 2017 to 2024 reveal significant variations in both food and inhalation allergen positivity rates (Table 7). Food allergen sensitization exhibited a substantial increase, rising from 1.67% in 2017 to 12.64% in 2023, followed by a decrease to 10.36% in 2024. Similarly, inhalation allergen sensitization rates increased from 2.41% in 2017 to 10.26% in 2023, with a subsequent decline to 8.33% in 2024. Notably, both allergen types demonstrated markedly elevated sensitization rates during the period from 2022 to 2024 compared to earlier years. Statistical analysis confirmed highly significant temporal variations for both food allergens (χ^2^ = 3914.486, *p* < 0.001) and inhalation allergens (χ^2^ = 2452.222, *p* < 0.001).

**Table 7:** Temporal trends in food and inhalation allergen sensitization rates (2017‒2024).

**Year**	**Food allergen**		**Inhalation allergen**	
	**n**	**%**	**n**	**%**
2017	76	1.67	165	2.41
2018	444	2.54	840	3.20
2019	344	2.14	803	3.34
2020	189	1.71	518	3.13
2021	254	1.61	866	3.66
2022	1,006	7.38	1,513	7.39
2023	2,146	12.64	2,614	10.26
2024	980	10.36	1,183	8.33
χ^2^	3914.486		2452.222	
P	0.000		0.000	

Note: The annual prevalence (%) for each allergen type is calculated as (Number of positive cases in that year / Total number of participants enrolled in that year) × 100%. Annual participant totals are provided in Table 1. The p-values result from Chi-Square tests for trend across all years.

### Age-specific and disease-specific sensitization patterns

Age-specific analysis (Table 8) revealed that food allergen sensitization was most prevalent among children under 5-years (6.69%) and those aged 5‒10 years (8.22%), with a decline observed as age increased. In contrast, inhalation allergen sensitization peaked in the 10‒20 years age group (10.92%) and remained elevated in younger cohorts before gradually decreasing. Disease-specific analysis (Table 9) indicated that asthma patients exhibited the highest rate of food allergen sensitization (6.21%), followed closely by allergic rhinitis (5.99%) and allergic purpura (5.76%). Regarding inhalation allergens, allergic rhinitis demonstrated the highest sensitization rate (6.77%), followed by allergic purpura (6.71%) and asthma (5.44%). Statistical analysis confirmed significant disease-specific variations for both allergen types (both p < 0.001).

**Table 8:** Age-specific prevalence of food and inhalation allergen sensitization.

**Age group**	**Food allergen**		**Inhalation allergen**	
	**n**	**%**	**n**	**%**
< 5	1,617	6.69	1,125	3.10
5‒10	1,167	8.22	1,907	8.96
10‒20	754	7.34	1,683	10.92
20‒30	658	3.60	1,416	5.17
30‒40	427	3.23	900	4.54
40‒50	276	3.30	612	4.88
50‒60	229	2.89	415	3.49
60‒70	199	3.51	291	3.43
≥70	112	3.84	153	3.49
χ^2^	858.538		2041.970	
p	0.000		0.000	

Note: ‘%’ represents the prevalence within each age group, calculated as (Number of positive cases in the age group / Total number of participants in that age group) × 100%. Age group totals are provided in Table 2.

**Table 9:** Disease-specific patterns of food and inhalation allergen sensitization.

**Disease**	**Food allergen**		**Inhalation allergen**	
	**n**	**%**	**n**	**%**
Dermatitis	399	3.08	929	4.79
Eczema	179	2.99	383	4.27
Urticaria	1,000	4.88	1,330	4.32
Asthma	1,717	6.21	2,255	5.44
Allergic rhinitis	1,029	5.99	1,744	6.77
Allergic purpura	887	5.76	1,550	6.71
Other	228	4.25	311	3.87
χ^2^	280.980		316.114	
p	0.000		0.000	

Note: ‘%’ represents the prevalence within each disease group, calculated as (Number of sensitized patients within the disease group / Total number of patients with that disease) × 100%. Disease group totals are provided in Table 3.

### Sub-analysis of higher levels of sensitization (sIgE ≥ 3.51 kUA/L)

To provide clinically more relevant information regarding IgE sensitization patterns, we performed a sub-analysis restricted to patients with “higher levels of sensitization” defined as sIgE ≥ 3.51 kUA/L (ImmunoCAP Class ≥ 3). As shown in Table 1, the patterns observed in this sub-analysis generally aligned with the primary findings but with notable differences in magnitude.

Among patients with higher levels of sensitization to food allergens, asthma continued to show the highest rate (2.84%), followed by allergic rhinitis (2.39%) and urticaria (1.87%). For inhalation allergens, allergic rhinitis maintained the highest rate (1.81%) of higher levels of sensitization, followed by allergic purpura (1.63%) and asthma (1.30%). Statistical analysis revealed significant disease-specific variations in the distribution of higher-level sensitization for both food allergens (χ^2^ = 89.456, *p* < 0.001) and inhalation allergens (χ^2^ = 112.234, *p* < 0.001). Compared to the primary analysis which included all sensitizations (≥ 0.35 kUA/L), the higher levels of sensitization rates were substantially lower, indicating that a considerable proportion of detected sensitizations were of low to moderate levels (Class 1‒2). This finding underscores the distinction between laboratory-detected sensitization and clinically relevant allergy, and highlights the importance of integrating *sIgE* level interpretation with clinical history for accurate diagnosis.

### Disease-specific sensitization patterns of major allergens

The distribution of specific allergen sensitization patterns across various allergic diseases revealed significant variations, as detailed in Table 10. *Egg white* exhibited the highest sensitization rates among asthma patients (19.57%), while *Artemisia argyi* demonstrated broad reactivity across multiple conditions, particularly in dermatitis (17.19%). *Cat hair allergen* was notably prevalent in allergic rhinitis (14.39%) and asthma (8.98%), while sensitivity to *milk allergen* was particularly elevated in the 'other' disease category (15.67%). Most allergens displayed statistically significant disease-specific variation patterns (*p* < 0.001). Notably, *Aspergillus fumigatus* showed no statistically significant variation across diseases (χ^2^ = 4.660, *p* = 0.588).

**Table 10:** Positive distribution of allergens in different diseases.

**Allergen Category**	**Allergen**	**Dermatitis**		**Eczema**		**Urticaria**		**Asthma**		**Allergic rhinitis**		**Allergic purpura**		**Other**		**χ^2^**	**p**
		**n**	**%**	**n**	**%**	**n**	**%**	**n**	**%**	**n**	**%**	**n**	**%**	**n**	**%**		
Common Food Allergens	Egg white	102	6.31	53	7.09	294	11.47	676	19.57	333	17.30	198	9.23	47	7.01	304.482	0.000
	Cow’s milk	68	4.21	20	2.67	133	5.19	240	6.95	133	6.91	158	7.36	105	15.67	135.696	0.000
	Peanut	68	4.21	38	5.08	214	8.35	290	8.40	176	9.14	149	6.94	17	2.54	71.529	0.000
	Wheat	25	1.55	8	1.07	39	1.52	61	1.77	89	4.62	58	2.70	3	0.45	82.259	0.000
	Soybean	16	0.99	4	0.53	29	1.13	82	2.37	92	4.78	69	3.22	3	0.45	111.582	0.000
	Shrimp	27	1.67	12	1.60	56	2.18	67	1.94	29	1.51	97	4.52	22	3.28	60.146	0.000
	Crab	12	0.74	2	0.27	14	0.55	32	0.93	16	0.83	33	1.54	20	2.99	44.868	0.000
	Almond	81	5.01	42	5.61	221	8.62	269	7.79	161	8.36	125	5.82	11	1.64	65.793	0.000
Perennial Indoor Allergens	House dust mite	65	4.02	15	2.01	82	3.20	122	3.53	110	5.71	165	7.69	52	7.76	99.263	0.000
	German cockroach	35	2.16	4	0.53	18	0.70	30	0.87	18	0.94	18	0.84	42	6.27	164.594	0.000
	Aspergillus fumigatus	2	0.12	2	0.27	6	0.23	10	0.29	2	0.10	8	0.37	1	0.15	4.660	0.588
	Candida albicans	17	1.05	11	1.47	29	1.13	47	1.36	39	2.03	45	2.10	9	1.34	13.897	0.031
	Dog dander	32	1.98	16	2.14	76	2.97	183	5.30	90	4.68	94	4.38	20	2.99	52.083	0.000
	Cat dander	61	3.77	20	2.67	75	2.93	310	8.98	277	14.39	261	12.16	23	3.43	344.811	0.000
Seasonal Aeroallergens	Mugwort	278	17.19	102	13.64	261	10.18	364	10.54	270	14.03	309	14.40	93	13.88	68.265	0.000
	Hop	23	1.42	13	1.74	40	1.56	207	5.99	220	11.43	111	5.17	3	0.45	345.295	0.000
	Ragweed	26	1.61	11	1.47	70	2.73	126	3.65	127	6.60	71	3.31	2	0.30	110.782	0.000
	Bermuda grass	136	8.41	82	10.96	271	10.57	351	10.16	209	10.86	188	8.76	31	4.63	32.388	0.000
	Timothy grass	190	11.75	91	12.17	318	12.41	376	10.89	233	12.10	171	7.97	9	1.34	94.360	0.000
	*Platanus* spp.	64	3.96	16	2.14	84	3.28	129	3.73	149	7.74	109	5.08	26	3.88	75.048	0.000

Data are presented as percentage of patients with positive sIgE (≥0.35 kUA/L) within each disease group. p-values were calculated by Chi-Square test across disease categories. Allergen names have been standardized to ImmunoCAP nomenclature; complete component information is available in Supplementary Table S1.

## Discussion

This large-scale, retrospective study provides a comprehensive analysis of *IgE* sensitization profiles among 13,123 patients diagnosed with allergic diseases in Northwest China. Our findings offer several key insights into the demographic factors shaping allergic sensitization within this clinical cohort.

The most prominent finding is the consistent and significant male predominance in sensitization rates for both food and inhalation allergens.^20^ This observation aligns with established evidence indicating sex-based differences in immune responses, often attributed to the modulating effects of sex hormones on immune cell function.^17^^,^^21^ Androgens may promote IgE synthesis through the upregulation of “IL-4” expression.^22^^,^^23^ The higher sensitization rates observed in males, particularly during early childhood, suggest a biological predisposition that should be considered in clinical risk assessment and early screening strategies.^24^^,^^25^

The age-specific patterns we observed reflect the classic “allergic march” paradigm, albeit with population-specific characteristics. Food allergen sensitization peaked in children aged 5‒10 years, significantly surpassing rates in older age groups. This highlights a critical developmental window and underscores the importance of targeted screening and dietary guidance in pediatric care.^12^ The subsequent rise in inhalation allergen sensitization during adolescence (10‒20 years) likely reflects cumulative environmental exposure and immune maturation, emphasizing the need for age-tailored allergen avoidance advice and patient education.

From a clinical perspective, the disease-specific sensitization patterns offer valuable insights. The substantial sensitization burden observed in patients with asthma and allergic rhinitis underscores the central role of IgE in these respiratory conditions, warranting clinician vigilance for polysensitization.^26^, ^27^, ^28^, ^29^, ^30^ Notably, peanut elicited the highest level of reactivity among food allergens, reinforcing its global status as a clinically significant and potent allergen.^13^^,^^31^

Critical Appraisal of Temporal Trends and IgE-Negative Cases: Our sub-analysis using a higher threshold of sIgE ≥ 3.51 kUA/L further supports this distinction. While the overall sensitization rates (≥ 0.35 kUA/L) for inhalation allergens in asthma and allergic rhinitis were modest at 5.4% and 6.8%, respectively, the rates of higher levels of sensitization (≥ 3.51 kUA/L) were considerably lower (1.3% and 1.8%, respectively). Notably, even at this higher threshold, the disease-specific variations remained statistically significant for both food and inhalation allergens (p < 0.001), suggesting that the observed disease-associated patterns are not merely driven by low-level, potentially clinically irrelevant sensitizations. This suggests that a substantial portion of sensitizations detected in this cohort are of low to moderate levels, reinforcing the principle that the detection of sIgE alone does not equate to clinical allergy, and that test results must be interpreted within the full clinical context. Taken together with the observation that nearly two-thirds (63.96%) of clinically diagnosed patients had no detectable *sIgE* to the tested panel (Table 5), our findings highlight a critical disconnect between clinical diagnosis and laboratory evidence of IgE-mediated sensitization, emphasizing the need for cautious interpretation of sIgE results within the full clinical context. We wish to address two key interpretative points raised during review, which are crucial for contextualizing our findings. First, the apparent temporal trend showing an increase in sensitization prevalence from 2017 to 2023 (Table 7) must be interpreted with considerable caution. Given the retrospective, single-center nature of this study, these fluctuations may not solely reflect true epidemiological shifts. They are likely confounded by evolving factors over the seven-year period, including changes in clinical awareness, diagnostic referral patterns, departmental testing protocols, and the implementation of updated guidelines influencing test selection. For instance, increased testing rates post-2020 may correlate with heightened focus on respiratory health. Therefore, we refrain from inferring a genuine temporal increase in population-level allergic sensitization. The primary value of this analysis lies in documenting the within-cohort testing patterns under real-world clinical conditions at our center over time. Second, the finding that 63.96% of patients with a clinical diagnosis of an allergic disease had no detectable serum sIgE to the tested panel (Table 5) is critically important and merits explicit discussion. This clearly underscores that a clinical diagnosis of “allergy” is not synonymous with IgE-mediated sensitization detectable by systemic serology. Several factors contribute to this discrepancy: 1) A significant proportion of clinically diagnosed conditions (e.g., certain forms of urticaria, allergic purpura/IgA vasculitis, some dermatitis cases) are driven by non-*IgE*-mediated mechanisms (e.g., immune complex, cell-mediated, or other inflammatory pathways). 2) Sensitization may be directed against allergens not included in our standard panel. This is particularly relevant given the relatively low sensitization rates observed for the tested inhalation allergens in patients with asthma (5.4%) and allergic rhinitis (6.8%) (Table 9), which may reflect the absence of key regional aeroallergens ‒ such as specific local pollens, molds, or other environmental proteins ‒ from our testing panel. 3) Serum sIgE levels may not capture localized IgE production in tissues (e.g., nasal mucosa in allergic rhinitis). While systemic immunosuppressants (e.g., oral corticosteroids) could potentially influence IgE synthesis over time, antihistamines and topical corticosteroids (inhaled, intranasal, or cutaneous) are not expected to suppress detectable serum IgE levels. Thus, medication use at the time of testing is unlikely to account for the high proportion of negative results. 4) The clinical diagnosis, made by a specialist based on history and examination, remains paramount; sIgE testing is a supportive, not definitive, tool. This finding reinforces the essential principle that the diagnosis and management should be guided primarily by the clinical picture, with IgE testing serving as an adjunct for identifying specific triggers in relevant cases, and highlights the critical need for regionally tailored allergen panels that reflect local exposure patterns.

It is worth noting that IgA vasculitis (Henoch-Schönlein purpura) is primarily a type III hypersensitivity disorder mediated by immune complex deposition, rather than an IgE-mediated condition. Despite this pathophysiological distinction, we elected to retain this subgroup in our analysis for two main reasons. First, in routine clinical practice in China, patients with IgA vasculitis are frequently evaluated in allergy clinics and undergo serum sIgE testing as part of their diagnostic workup, reflecting the real-world diagnostic classification of “allergic purpura”. Second, although the underlying mechanism differs, exploring sIgE sensitization patterns in this population may still provide clinically relevant information, as atopic background and IgE sensitization have been reported to be associated with disease severity or recurrence in some studies.^32^^,^^33^ In the present cohort, patients with allergic purpura exhibited measurable IgE sensitization to both food and inhalation allergens, with a rate of higher-level sensitization (sIgE ≥ 3.51 kUA/L) for inhalation allergens (1.63%) that was comparable to, or even exceeding, that observed in classical IgE-mediated conditions such as asthma (1.30%). Although the underlying pathophysiology of IgA vasculitis is distinct from IgE-mediated immediate hypersensitivity, this finding lends indirect support to the hypothesis that an atopic background may modulate disease expression.

## Limitations

Our study has several limitations. First, its single-center, retrospective design may affect the generalizability of findings and as discussed, complicates the interpretation of temporal trends. Second, we defined sensitization by an sIgE level ≥ 0.35 kUA/L. It is vital to reiterate that sensitization is not equivalent to clinical allergy; our prevalence data are derived from a cohort with clinician-diagnosed disease, providing necessary context, but the clinical relevance of specific sensitizations requires confirmation by history and challenge tests. Furthermore, the relatively low sensitization rates to inhalation allergens among patients with asthma (5.4%) and allergic rhinitis (6.8%) suggest that our standard panel may have lacked coverage of important regional aeroallergens prevalent in Northwest China. This limitation underscores the need for future studies to incorporate comprehensive, locally-relevant allergen panels, potentially informed by aerobiological monitoring and regional sensitization mapping. Third, data collection relied on clinical records, which may introduce documentation bias. Fourth, we fully recognize the mechanistic heterogeneity within our cohort. The inclusion of non-IgE-mediated conditions such as IgA vasculitis (allergic purpura) represents a limitation, as the underlying pathophysiology differs from classical IgE-mediated allergic diseases. Future research should analyze IgE-mediated and non-IgE-mediated allergic diseases separately to delineate their distinct sensitization profiles. Future multi-center, prospective studies incorporating detailed symptom phenotyping and component-resolved diagnostics are needed to validate and extend these findings.^25^^,^^34^

## Conclusions and implications

In conclusion, this study delineates clear influences of sex and age on IgE sensitization patterns within a large Chinese clinical cohort. It provides evidence for higher sensitization rates in males and an age-dependent shift from food to inhalation allergen predominance. These findings advocate for a more stratified approach to allergy management. Key clinical implications include: 1) Considering early screening in young males at higher risk, 2) Implementing pre-seasonal prophylaxis based on regional pollen calendars, and 3) Tailoring patient education to age-specific risks. Ultimately, this work contributes a detailed sensitization profile from Northwest China, establishing a foundation for advancing personalized allergy care while highlighting the complex interplay between clinical diagnosis and laboratory findings in allergic diseases. Furthermore, our sub-analysis using a higher sIgE threshold (≥3.51 kUA/L) revealed that even among sensitized patients, the majority exhibited low-to-moderate level reactivity, underscoring that laboratory-detected sensitization does not equate to clinically relevant allergy. Importantly, the substantial proportion of IgE-negative results among clinically diagnosed patients ‒ particularly the low sensitization rates to inhalation allergens in asthma and allergic rhinitis ‒ highlights the critical need for regionally customized diagnostic panels that include locally prevalent aeroallergens. Future research should prioritize the development and validation of such panels, informed by aerobiological data and component-resolved diagnostics, to improve the detection of clinically relevant sensitizations in diverse populations.

## Authors’ contributions

Jiahao Guan: Conceptualization; data curation; formal analysis; funding acquisition; resources; writing- original draft; writing-review and editing.

Fei Wang: Data curation; formal analysis; writing-original draft; writing-review and editing.

Zitong Wang: Data curation; formal analysis; writing-original draft; writing-review and editing.

Yihan Dong: Data curation; writing-original draft; writing- review and editing.

Jun Wang: Data curation; formal analysis.

Yajuan Ren: Data curation; formal analysis.

Zifan Lu: Writing-original draft; writing-review and editing.

Lixia Zhang: Conceptualization; data curation; formal analysis; methodology; funding acquisition.

## Ethics approval and informed consent

The study protocol was approved by the Ethics Committee of Shaanxi Provincial People’s Hospital [Approval No. (2024) Ethics Review No. (R131), Approval date: June 13, 2024], and is a retrospective analysis. In this study, informed consent from patients is not required. The patient data in this research have been anonymized, and sensitive personal information and commercial interests are not involved in the data processing and analysis. According to the relevant legal provisions of the “Notice on Issuing the Measures for Ethical Review of Life Science and Medical Research Involving Humans” (Guowei Kejiaofa [2023] No. 4), this study meets the situation specified in Point (ii) of Article 32, Chapter III of the Measures, and is exempted from ethical review.

## Declaration of generative AI and AI-assisted technologies in the manuscript preparation process

During the preparation of this work the authors used DeepSeek in order to improve language and readability. After using this tool, the authors reviewed and edited the content as needed and take full responsibility for the content of the published article.

## Funding

10.13039/501100013064Key Research and Development Program of Shaanxi (No. 2024SF-YBXM-110). The sponsor Lixia Zhang contributed to study conception and design.

## Data availability

The data that support the findings of this study are available from the corresponding author upon reasonable request.

## Declaration of competing interest

The authors declare that they have no known competing financial interests or personal relationships that could have appeared to influence the work reported in this paper.

## Appendix Supplementary materials

Image, application 1

Image, application 2

Image, application 3
