# Supplementary material for: Population-specific patterns of IgE sensitization in Northwest China
Source: Clinics (Sao Paulo). 2026 Sep 8;81:101121. doi: 10.1016/j.clinsp.2026.101121 (PMC13573707; doi:10.1016/j.clinsp.2026.101121)
Supplement: Supplementary file 1 [file mmc1.docx]

陕西省人民医院伦理委员会	编 号 ：S	P	P H - L L B G -	1	7 - 3	. 2

**陕西省人民医院医学伦理委员会科学技术研究项目审批件**

Approval Letter of Medical Ethics Comnittee of Shaanxi Provincial People’s Hospital

(2024)伦审第 (R131) 号

| 项目名称 | 宏基因组二代测序分析及诊断技术在重症社区获得性肺炎患者下呼吸道病原 学早期诊断中的应用及其研究价值 | | | | | | |
| --- | --- | --- | --- | --- | --- | --- | --- |
| 项目来源 | □国家计划  □国际合作 | | □部委计划 □省、市计划  □其它单位委托 □自选 | | | ■基金资助 □其它 | |
| 计划(基金)编号 | S2024-YF-YBSF-0702 | | | | | | |
| 主要研究者及专业 | 王君/呼吸与危重症一科 | | | | | | |
| 参加研究人员 | 张薇，李博玲，邓丽娟等 | | | | | | |
| 审查方式 | □会议审查 ■ 快速审查 口备案 | | | | | | |
| 审查文件 | 1.项目申请书  2.免除知情同意申请  3.立项批准文件  4.研究材料诚信承诺书 | | | | | | |
| 审查结论 | ■同意 □作必要修正后同意 □作必要修正后重审  □不同意 □终止或暂停已经批准的临床试验 | | | | | | |
| 审批意见 | | | | | | | |
| 根据《涉及人体的医学科学技术研究管理办法(征求意见稿)》、《生物医学新技术临床应用  管理条例(征求意见稿)》和《涉及人的生命科学和医学研究伦理审查办法》,伦理委员会审查后  认为，研究方案设计科学合理，对受试者保护措施得当，主要研究者具有开展临床试验的相应资质 和能力，同意该项目开展临床研究。 | | | | | | | |
| 联系人 | | 王卓 | | 联系电话 | 029-85251331-2610 | | |
| 地 址 | | 陕西省西安市友谊西路256号 | | | 邮 编 | | 710068 |
| 主任委员签字 | | 微气 | | | 审批日期 | | 2024年6月13日 |
| 伦理委员会 | | 陕西省人民医院医学伦理委员会 (盖章) | | | | | |
