# Supplementary material for: Population-specific patterns of IgE sensitization in Northwest China
Source: Clinics (Sao Paulo). 2026 Sep 8;81:101121. doi: 10.1016/j.clinsp.2026.101121 (PMC13573707; doi:10.1016/j.clinsp.2026.101121)
Supplement: Supplementary file 2 [file mmc2.docx]

**CLINICS-D-26-00972_Supplementary Material**

**Supplementary Table S1** Comprehensive list of allergens tested, with standardized nomenclature, common names, scientific names, and ImmunoCAP codes.

| **Standardized Name (This Study)** | **Common / Variant Names Encountered** | **Scientific Name (Species)** | **Allergen Type** | **ImmunoCAP Allergen Code (Example)** | **Notes / Clarification** |
| --- | --- | --- | --- | --- | --- |
| INHALANT ALLERGENS |  |  |  |  |  |
| Artemisia argyi | Mugwort, Artemisia annua | Artemisia argyi | Pollen (Weed) | w231 | Predominant mugwort species in Northwest China. |
| Humulus scandens | Humulus, Japanese hop | Humulus scandens | Pollen (Weed) | w203 | Common regional weed pollen. |
| Platanus spp. | Platanus, Plane tree | Platanus acerifolia (common) | Pollen (Tree) | t7 | Common urban tree pollen. |
| Timothy grass | Pasture, Grass, Forage grass | Phleum pratense | Pollen (Grass) | g6 | Representative of common grass pollen mix. |
| Ragweed | – | Ambrosia artemisiifolia | Pollen (Weed) | w1 |  |
| Bermuda grass | Bermudagrass | Cynodon dactylon | Pollen (Grass) | g2 |  |
| House dust mite (D. pteronyssinus) | Acaroid mite, Claw mite, Claw free mite | Dermatophagoides pteronyssinus | Indoor (Mite) | d1 | Major household mite allergen. |
| House dust mite (D. farinae) | Acaroid mite, Claw mite | Dermatophagoides farinae | Indoor (Mite) | d2 | Major household mite allergen. |
| Cat dander | Cat hair | Felis domesticus | Animal | e1 |  |
| Dog dander | Dog hair | Canis familiaris | Animal | e5 |  |
| German cockroach | Cockroach | Blattella germanica | Insect | i6 |  |
| Aspergillus fumigatus | – | Aspergillus fumigatus | Mold (Fungus) | m3 |  |
| Candida albicans | – | Candida albicans | Yeast (Fungus) | m5 |  |
| FOOD ALLERGENS |  |  |  |  |  |
| Egg white | Egg | Gallus domesticus | Food | f1 |  |
| Cow’s milk | Milk | Bos domesticus | Food | f2 |  |
| Peanut | – | Arachis hypogaea | Food | f13 |  |
| Shrimp | – | Penaeus spp. | Food | f24 |  |
| Crab | – | Charybdis spp. | Food | f23 |  |
| Wheat | – | Triticum aestivum | Food | f4 |  |
| Soybean | Soy | Glycine max | Food | f14 |  |
| Almond | – | Prunus dulcis | Food | f20 |  |

Note: Nomenclature is standardized according to the ImmunoCAP system (Thermo Fisher Scientific). ‘Common/Variant Names Encountered’ are included for reference but were harmonized to the ‘Standardized Name’ for all analyses.
