# Supplementary material for: Population-specific patterns of IgE sensitization in Northwest China
Source: Clinics (Sao Paulo). 2026 Sep 8;81:101121. doi: 10.1016/j.clinsp.2026.101121 (PMC13573707; doi:10.1016/j.clinsp.2026.101121)
Supplement: Supplementary file 3 [file mmc3.docx]

**Ethics Approval**

The study protocol was approved by the Ethics Committee of Shaanxi Provincial People’s Hospital [Approval No. (2024) Ethics Review No. (R131)], and is a retrospective analysis. In this study, informed consent from patients is not required. The patient data in this research have been anonymized, and sensitive personal information and commercial interests are not involved in the data processing and analysis. According to the relevant legal provisions of the “Notice on Issuing the Measures for Ethical Review of Life Science and Medical Research Involving Humans” (Guowei Kejiaofa [2023] No. 4), this study meets the situation specified in Point (ii) of Article 32, Chapter III of the Measures, and is exempted from ethical review.
